# Efficacy of *16S rRNA* variable regions high-resolution melt analysis for bacterial pathogens identification in periprosthetic joint infections

**DOI:** 10.1186/s12866-021-02164-8

**Published:** 2021-04-13

**Authors:** Samaneh Bourbour, Mohammad Emaneini, Mahmoud Jabalameli, Seyed Mohammad Javad Mortazavi, Mohamad Naghi Tahmasebi, Amirheckmat Taghizadeh, Arash Sharafatvaziri, Reza Beigverdi, Fereshteh Jabalameli

**Affiliations:** 1 Department of Microbiology, School of Medicine, Tehran University of Medical Sciences, Tehran, Iran; 2 Department of Orthopedic Surgery, Shafa Yahyaiyan Hospital, Iran University of Medical Sciences, Tehran, Iran; 3 Department of Orthopedic Surgery, Imam Khomaini Hospital, Tehran University of Medical Sciences, knee and hip surgeon, Tehran, Iran; 4 School of Electrical and Computer engineering, college of engineering, University of Tehran, Tehran, Iran

**Keywords:** Periprosthetic joint infections, Pathogens, Broad-range PCR, High-resolution melt analysis

## Abstract

**Background:**

Accurate and rapid identification of microorganisms causing periprosthetic joint infections (PJIs) are necessary for choosing an appropriate antibiotic therapy. Therefore, molecular techniques are suggested for diagnosis in suspected PJIs. The Broad-range PCR and High-Resolution Melt Analysis (HRMA) were evaluated for the identification of causative organisms of PJIs in this study.

**Results:**

For 47 of 63 specimens, both the culture and broad-range PCR were positive. The culture was found to be able of organism’s detection in 74.6% (47/63) of patients. Of 47 positive cultures, 11 (23.4%) were polymicrobial and 36 (76.59%) were monomicrobial cultures, in which 34 (91.89%) cases were detected by HRM assay. The sensitivity, specificity of HRMA vs monomicrobial culture were 91.89, 93.75%, respectively. The sensitivity, specificity of total HRMA (mono + poly) vs culture were 82.92, 93.75%.

**Conclusions:**

HRM assay coupled with broad-range PCR are effective screening, rapid, and relatively cost-effective methods for discrimination of PJIs especially in aiding culture method. Using computer programs such as the Matlab-2018b program for HRM data analysis is also valuable and helpful in diagnosis.

## Background

Periprosthetic joint infections (PJIs), are among important threats to global public health and are considered as a main challenge after elective surgical procedures [1, 2]. Arthroplasty is a frequent surgical procedure for joint replacement in end-stage arthritis [3, 4]. One of the most serious and devastating complications after joint arthroplasty is PJI [5, 6]. Also, higher revision arthroplasty rates could be due to PJI [7].

Prevalence of PJI is steadily increasing, mostly due to more frequent usage of prosthetic joints [1, 2]. Given the projected increase of joint replacement over the coming decades turned into a challenging issue, such as diagnosis challenges, high morbidity, the economic burden for patients and healthcare costs [5, 8, 9]. In managing PJI, one of the most important aspects is a prompt and definitive diagnosis of the causative organisms for the selection of appropriate treatment options and avoiding unnecessary multiple surgical procedures. In up to 50% of PJI cases, the infecting organism is not isolated in cultures and the risk of reinfection in negative culture cases is 4.5 times higher than in positive culture cases [10–12].

Since most of the methods for the detection of causative bacteria are time-consuming and costly, several fast and accurate molecular techniques have been investigated to improve the sensitivity of PJI diagnosis [13]. Therefore, molecular techniques such as broad-range PCR targeting the *16SrRNA* gene (broad-range PCR) and high-resolution melt curve analysis (HRMA) of 16 s hypervariable gene regions contribute to PJIs rapid diagnosis. The Full-length *16S rRNA* gene presents in all bacteria and is including nine hypervariable regions (V1-V9). Broad-range PCR identify non-viable bacterial DNA in specimens whose culture are negative because of consuming antibiotics [14, 15].

The HRMA is as post-PCR technique with amplified on PCR amplicons and there is a relation between temperature and extent of double-stranded DNA denaturation. In this technique, %G-C content, amplicons length (<300 bp) and the nucleotide sequence contribute to melting temperature (Tm) and fluorescent dye binding to double-stranded DNA in the PCR reaction. In this method, as the temperature increases, the double-stranded DNA detaches into single-strands and leading to a reduction in fluorescence intensity. The advantages of this approach can be mentioned to rapid-speed, relatively cost-effective, use of generic instrumentation accessible in many laboratories, simplicity, single step, and an alternative to gel-based techniques [16–19].

An 18-month study was performed on patients with knee and hip PJIs from tertiary care hospitals. In this research, the infecting organisms were identified using cultures and phenotypic methods, broad-range PCR, HRMA in synovial fluid and tissue specimens from patients with suspected knee and hip PJIs. The raw data of HRMA were translated and analyzed by the Matlab-2018b program. To our knowledge, there have been no studies evaluating *16S rRNA* gene variable regions HRMA to identify the causative organisms in PJIs.

## Results

Over the study period, 63 patients (42 women and 21 men) with suspected knee or hip PJIs, and criteria included, were analyzed. Of 63 patients undergoing total arthroplasty, 33 knee and 30 hip were involved.

The specimens consisted of 39 synovial fluids and 24 tissues. Among the total 63 patients who were investigated, 47 (74.6%) had positive culture, while in 16 (25.38%) the cultures were negative. Of 47 positive cultures, the most common causative pathogen was *Staphylococcus aureus* (*S. aureus*, 17 cases, 36.17%) followed by coagulase-negative staphylococci (CoNS, 14 cases, 29.78%). Methicillin-resistant *S. aureus* (MRSA) was isolated in 5 cases (10.63%) and methicillin-resistant coagulase-negative staphylococci (MR-CoNS) were isolated in seven cases (14.89%). No strains were found vancomycin-resistance among Methicillin-resistant *Staphylococcus* spp. (MRS spp) isolates by E-test.

**Fig. 1:**
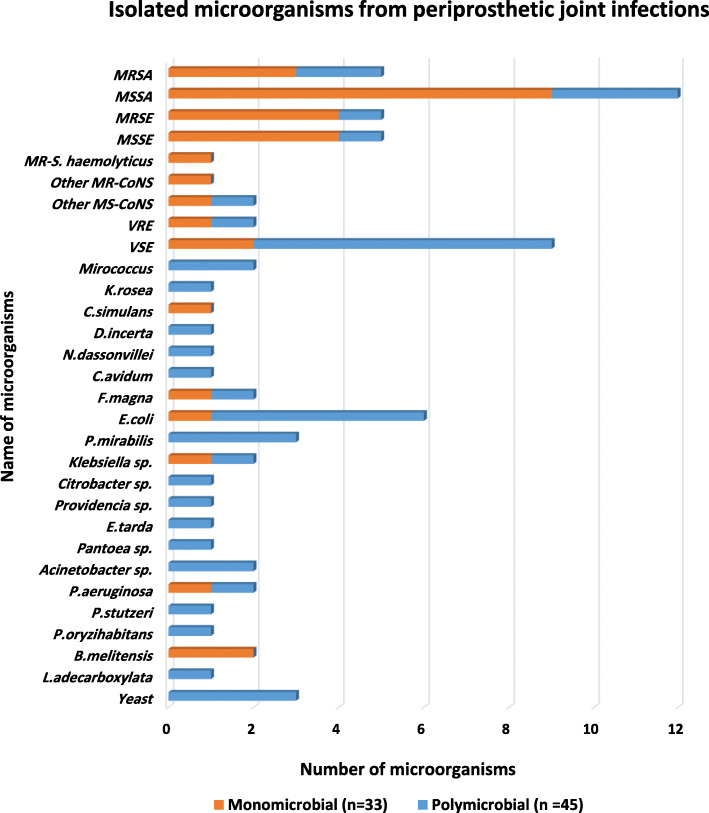
Distribution of detected microorganisms from PJIs MRSA: Methicillin-resistant *Staphylococcus aureus,* MSSA: Methicillin-susceptible *Staphylococcus aureus*, MRSE: Methicillin-resistant *Staphylococcus epidermidis*, MSSE: Methicillin-susceptible *Staphylococcus epidermidis*, MR-*S. haemolyticus:* Methicillin-resistant *Staphylococcus haemolyticus*, Other MR-CoNS: Other Methicillin-Resistant Coagulase-Negative Staphylococci, Other MS-CoNS: Other Methicillin-susceptible Coagulase-Negative Staphylococci, VSE*:* Vancomycin-sensitive *Enterococcus faecalis*, VRE: Vancomycin-resistant *Enterococcus faecalis*, C.*simulans*: *Corynebacterium simulans*, *D.incerta: Desemzia incerta, N.dassonville: Nocardiopsis dassonville, C.avidum: Cutibacterium avidum, F.magna*: *Finegoldia magna*, *B*.*melitensis*: *Brucella melitensis*, *E.coli*: *Escherichia coli, P.mirabilis: Proteus mirabilis, E.tarda: Edwardsiella tarda, P.aeruginosa: Pseudomonas aeruginosa, P.stutzeri: Pseudomonas stutzeri, P.oryzihabitans: Pseudomonas oryzihabitans, L.adecarboxylata: Leclercia adecarboxylata*

Of 47 positive cultures, 36 (76.59%) infections were monomicrobial and 11 (23.4%) were polymicrobial. In monomicrobial PJIs, the most common isolated bacterium was *S. aureus* (12/47, 25.53%) and several Gram-negative bacterial strains were isolated from specimens, such as *Escherichia coli, Klebsiella pneumonia, Pseudomonas aeruginosa.* Also, two isolates of *Brucella melitensis* were detected from monomicrobial PJIs. *Enterococcus faecalis* (11/47, 23.4%) isolates were the most commonly identified species in polymicrobial PJIs which one isolate was vancomycin-resistant enterococci (VRE). *Escherichia coli* (6/47, 12.76%) isolates were the most frequent isolate of Gram-negative in polymicrobial PJIs. Anaerobic pathogens as *Finegoldia magna* (*F. magna*) and *Cutibacterium avidum* (*C. avidum*) were isolated in three cases (5.45%) in mono and polymicrobial PJIs which are shown in Fig. 1. Broad-range PCR was performed on 63 specimens of which 47 specimens were positive. Overall, the results of culture and broad-range PCR were not in agreement with the two cases. There was one case of positive broad-range PCR in which the culture was negative and *F. magna* was detected with sequencing method. Furthermore, there was one case of culture-positive and *S. aureus* was identified in which broad-range PCR did not detect the organism.

**Fig. 2:**
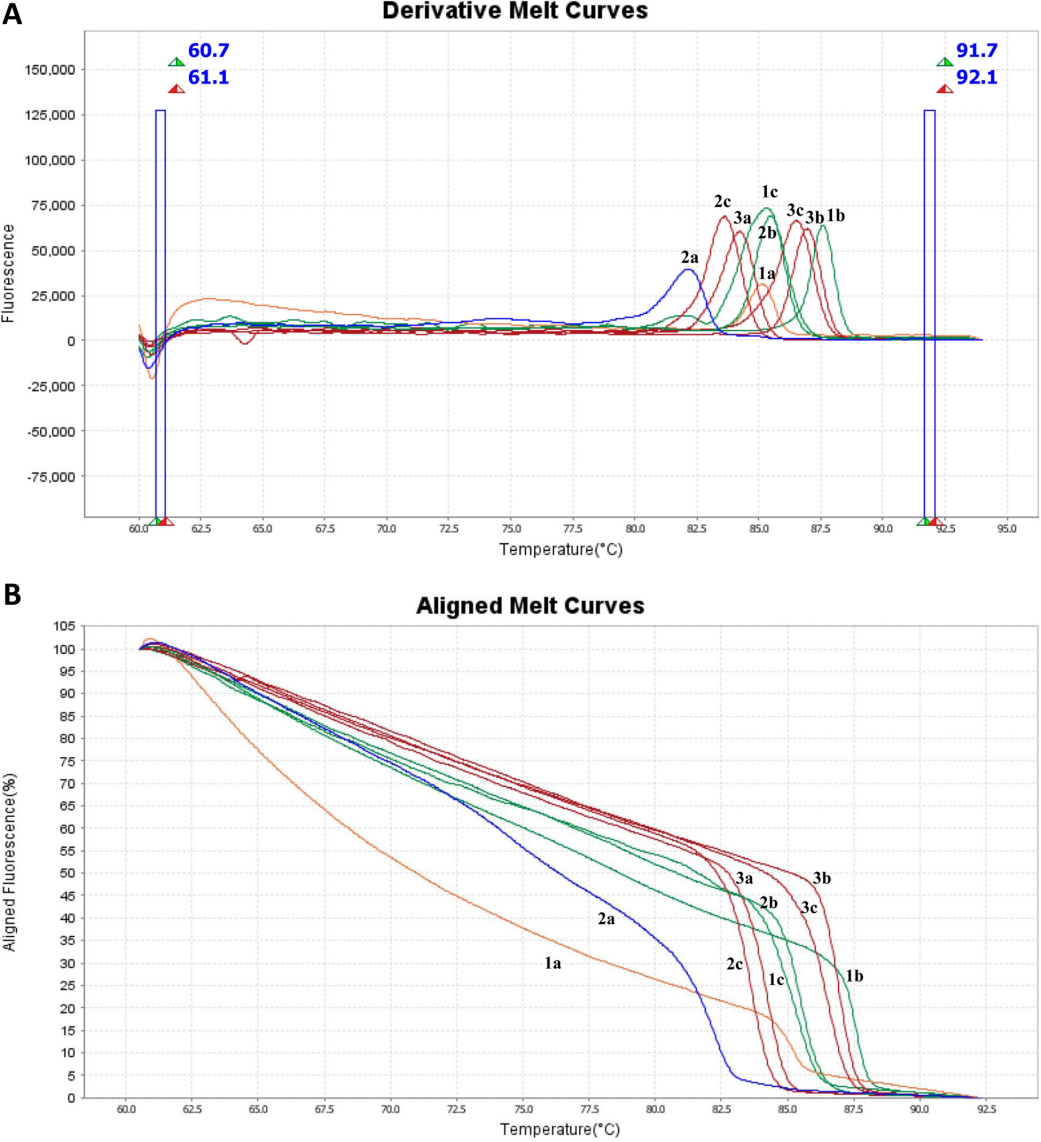
**A:** HRMA Derivative Melting Curves and **B:** HRMA Aligned Melting Curves for some of Reference strains **1a:** V1 region of *K. pneumonia* ATCC 700603, **1b:** V3 region of *K. pneumonia* ATCC 700603, **1c:** V6 region of *K. pneumonia* ATCC 700603, **2a:** V1 region of *S. aureus* ATCC 25923*,* **2b:** V3 region of *S. aureus* ATCC 25923, **2c:** V6 region of *S. aureus* ATCC 25923, **3a:** V1 region of *B. melitensis* 16 M ATCC 23456, **3b:** V3 region of *B. melitensis* 16 M ATCC 23456, **3c:** V6 region of *B. melitensis* 16 M ATCC 23456

**Fig. 3:**
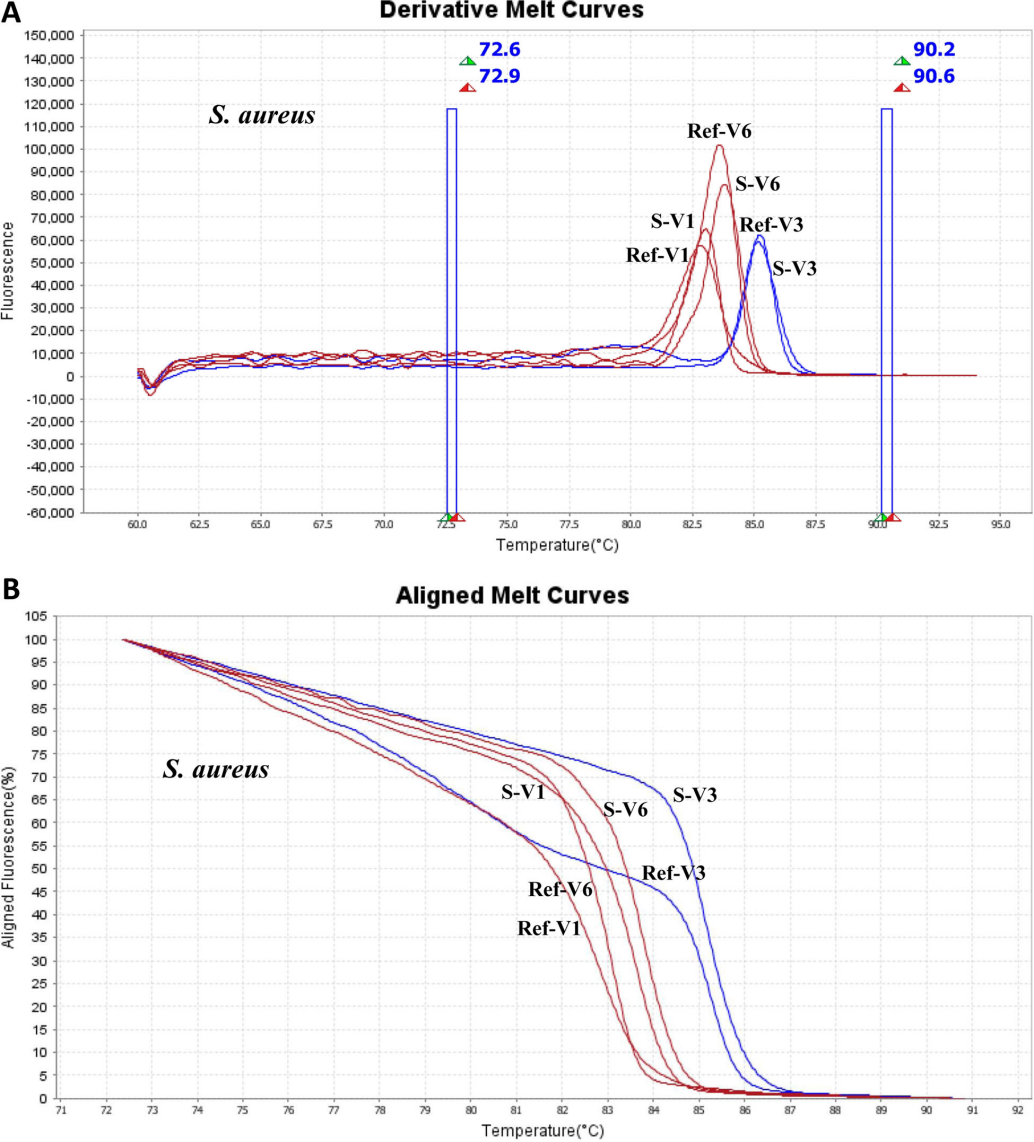
**a**: HRMA Derivative Melting Curves and **b**: HRMA Aligned Melting Curves for V1, V3, V6 regions of *S. aureus* ATCC 25923 and *S. aureus* isolated from clinical (PJI) specimens Ref-V1: Reference strain-V1 region, S-V1: Clinical specimen-V1 region, Ref-V3: Reference strain-V3 region, S-V3: Clinical specimen-V3 region, Ref-V6: Reference strain-V6 region, S-V6: Clinical specimen- V6 region

At first, a reference library based on Tm and melting curves of 19 reference strains was established by HRMA for detection of pathogens. Derivative and Aligned Melt Curves of three strains (*Klebsiella pneumonia* ATCC 700603, *S. aureus* ATCC 25923 and *Brucella melitensis*) with three *16SrRNA* regions are outlined in Fig. 2. The generated melt curves of samples (48 synovial fluid and tissue specimens) were compared with melt curve database of reference strains to perform bacterial identification that are shown in Fig. 3. The causative organisms were identified by HRMA in all 47 cases which broad-range PCR were positive (36 monomicrobial and 11 polymicrobial infections). Among 37 detected monomicrobial infections by HRMA, 34 (91.89%) cases were concordant with culture in which the four cases were accordant at the genus level and three (8.1%) cases were discordant with culture. The sensitivity, specificity, accuracy, Positive predictive values (PPV) and Negative predictive (NPV) of HRMA vs culture for detecting the monomicrobial infections in PJI samples were 91.89, 93.75, 97.14, 83.33, and 92.45%, respectively.

From 11 remaining case with a polymicrobial positive culture, HRMA was able to detect 7 cases (63.63%) at least with one of the causative bacterial species (one case was identified at the genus level) that were concordance to culture findings (Table 1). The Overall sensitivity, specificity, PPV and NPV, and accuracy of total HRMA (mono + poly) vs culture were 82.92, 93.75, 97.14, 68.18, and 85.96%, respectively.

**Table 1:** HRMA results of patient’s synovial fluids and tissues with three *16srRNA* gene hypervariable regions primers (Temp_Min:80/90, Temp_Max:89/90)

Samples	Detected Bacteria					
	Culture results		Broad-range PCR	HRMA ^**b**^ (Tm (°C) / Aggregated Difference or Aggregated Distance)		
	Monomicrobial / Polymicrobial ^**a**^	Microorganisms name				
				V1	V3	V6
**P1**	M / -	*S. aureus*	+	*S. aureus* (81.60/0.7)	*S. aureus* (84.10/0.1)	*S. aureus* (83.70/0.5)
**P2**	M / -	*S. aureus*	+	*S. aureus* (84.20/0.3)	*S. aureus* (85.10/0.1)	*S.aureus* (83.40/0.2)
**P3**	M / -	*S. aureus*	+	*S. aureus* (84.30/0.4)	*S. aureus* (85.10/0.1)	*S. aureus* (83.00/0.2)
**P4**	M / -	*S. aureus*	+	*S. aureus* (80.90/0.0)	*S. aureus* (84.00/0.0)	*S. aureus* (83.20/0.0)
**P5**	M / -	*S. aureus*	–	*S. aureus* (84.70/0.8)	*S. aureus* (84.40/0.6)	*S. aureus* (83.70/0.5)
**P6**	M / -	*S. aureus*	+	*S. aureus* (83.20/0.2)	*S. aureus* (84.90/0.1)	*S. aureus* (81.70/0.2)
**P7**	M / -	*S. epidermidis*	+	*S. epidermidis* (83.50/0.2)	*S. epidermidis* (85.10/0.4)	*S. epidermidis* (83.70/0.1)
**P8**	M / -	*S. epidermidis*	+	*S. epidermidis* (82.70/0. 2)	*S.epidermidis* (85.80/0.2)	*S.epidermidis* (83.80/0.2)
**P9**	M / -	*E. faecalis*	+	*S. epidermidis* (83.10/0.6)	*S. epidermidis* (85.60/0.0)	*S. epidermidis* (83.20/0.4)
**P10**	M / -	*E. faecalis*	+	*E. faecalis* (86.30/0.1)	*E. faecalis* (85.80/0.3)	*E. faecalis* (84.10/0.0)
**P11**	M / -	*S. haemolyticus*	+	*S. haemolyticus* (83.80/0.1)	*S. haemolyticus* (87.50/0.0)	*S. haemolyticus* (83.80/0.5)
**P12**	M / -	*K. pneumoniae*	*+*	*K. pneumoniae* (86.13/0.03)	*K. pneumoniae* (86.80/0.7)	*K. pneumoniae* (82.95/0.5)
**P13**	M / -	*E. coli*	+	*E. coli* (85.40/0.4)	*E. coli* (86.20/0.1)	*E. coli* (84.50/0.3)
**P14**	M / -	*P. aeruginosa*	+	*P. aeruginosa* (87.50/0.5)	*P. aeruginosa* (85.30/0.8)	*P. aeruginosa* (84.90/0.1)
**P15**	M / -	*B. melitensis*	+	*B. melitensis* (82.60/0.0)	*B. melitensis* (85.40/0.1)	*B. melitensis* (86.40/0.4)
**P16**	M / -	*B. melitensis*	+	*B. melitensis* (82.70/0.1)	*B. melitensis* (85.40/0.1)	*B. melitensis* (86.40/0.4)
**P17**	- / -	–	+	*F. magna* (84.30/0. 0)	*F. magna* (85.50/0.0)	*F. magna* (82.20/0.0)
**P18**	M / -	*S. aureus*	+	*S. epidermidis* (83.20/0.5)	*S. epidermidis* (85.20/0.3)	*S. epidermidis* (83.50/0.3)
**P19**	M / -	*S. aureus*	*+*	*S. epidermidis* (83.10/0.6)	*S. epidermidis* (85.20/0.3)	*S. epidermidis* (83.80/0.0)
**P20**	M / -	*S. aureus*	+	*S. haemolyticus* (83.70/0.2)	*S. haemolyticus* (85.90/0.0)	*S. haemolyticus* (83.40/0.3)
**P21**	M / -	*S. epidermidis*	+	*S. epidermidis* (83.90/0.2)	*S. epidermidis* (85.70/0.2)	*S. epidermidis* (83.90/0.1)
**P22**	M / -	*S. aureus*	+	*S. aureus* (83.40/0.3)	*S. aureus* (85.50/0.0)	*S. aureus* (83.20/0.5)
**P23**	M / -	*S. aureus*	+	*S. aureus* (80.94/0.54)	*S. aureus* (85.80/0.54)	*S. aureus* (82.56/0.54)
**P24**	M / -	*S. aureus*	+	*S. aureus* (80.94/0.04)	*S. aureus* (84.10/0.1)	*S. aureus* (82.80/0.4)
**P25**	M / -	*S. epidermidis*	+	*S. epidermidis* (82.30/0.6)	*S. epidermidis* (84.30/1.0)	*S. epidermidis* (84.10/0.4)
**P26**	M / -	*S. epidermidis*	+	*E. faecalis* (86.10/0.3)	*E. faecalis* (84.20/0.7)	*E. faecalis* (82.80/0.18)
**P27**	M / -	*S. epidermidis*	*+*	*S. epidermidis* (83.20/0.6)	*S. epidermidis* (84.20/0.22)	*S. epidermidis* (83.60/0.81)
**P28**	M / -	*S. aureus*	+	*S. aureus* (82.10/1.2)	*S. aureus* (83.80/0.2)	*S. aureus* (83.20/0.0)
**P29**	M / -	*S. epidermidis*	+	*E. faecalis* (86.30/0.1)	*E. faecalis* (85.80/0.3)	*E. faecalis* (84.00/0.1)
**P30**	M / -	*S. epidermidis*	*+*	*S. epidermidis* (80.90/0.8)	*S. epidermidis* (85.50/0.2)	*S. epidermidis* (84.20/0.5)
**P31**	M / -	*S. aureus*	*+*	*S. aureus* (84.20/0.3)	*S. aureus* (85.50/0.5)	*S. aureus* (83.20/0.0)
**P32**	M / -	*S. aureus*	*+*	*S. epidermidis* (81.70/0.0)	*S. epidermidis* (84.10/1.2)	*S. epidermidis* (83.80/0.1)
**P33**	M / -	*S. aureus*	*+*	*S. aureus* (83.50/0.4)	*S. aureus* (80.90/0.04)	*S. aureus* (82.40/0.1)
**P34**	M / -	*E. faecalis*	+	*E. faecalis* (85.89/0.51)	*E. faecalis* (85.47/0.53)	*E. faecalis* (83.73/0.07)
**P35**	M / -	*S. epidermidis*	+	*S. epidermidis* (82.60/0.1)	*S. epidermidis* (86.10/0.5)	*S. epidermidis* (83.00/0.6)
**P36**	M / -	*S .aureus*	+	*S. aureus* (81.50/0.2)	*S. aureus* (86.10/0.3)	*S. aureus* (83.10/0.5)
**P37**	M / -	*E. faecalis*	+	*E. faecalis* (86.51/0.1)	*E. faecalis* (81.98/4.02)	*E. faecalis* (83.96/0.2)
**P1**	- / P	*S. epidermidis, A. baumannii*	+	*F. magna* (85.50/1.0)	*F. magna* (85.20/0.3)	*F. magna* (82.90/0.2)
**P2**	P / -	*S. aureus, P. mirabilis*	+	*S. sanguinis* (83.60/0.1)	*S. sanguinis* (86.30/0.1)	*S. sanguinis* (83.90/0.1)
**P3**	P / -	*S. aureus, C. avidum*	+	*C. avidum* (85.80/0.1)	*C. avidum* (89.00/0.1)	*C. avidum* (85.60/0.3)
**P4**	P / -	*S. aureus, E. faecalis*	+	*S. aureus* (83.90/0.2)	*S. aureus* (85.90/0.4*)*	*S. aureus* (83.10/0.4)
**P5**	P / -	*F. magna*, *E. faecalis*	+	*F. magna* (84.20/0.3)	*F. magna* (85.30/0.2)	*F. magna* (82.70/0.0)
**P6**	P / -	*E .faecalis, P. mirabilis*	+	*F. magna* (84.20/0.3)	*F. magna* (86.80/1.3)	*F. magna* (82.60/0.1)
**P7**	P / -	*S. epidermidis*, yeast	+	*S. aureus* (80.90/0.0)	*S. aureus* (84.80/0.8)	*S. aureus* (83.60/0.4)
**P8**	P / -	*E. faecalis, P. mirabilis*	+	*S. aureus* (80.90/0.0)	*S. aureus* (83.30/0.7)	*S. aureus* (82.10/1.1)
**P9**	P / -	*S. aureus, P. mirabilis*	+	*P. mirabilis* (83.50/0.3)	*P. mirabilis* (87.00/0.2)	*P. mirabilis* (84.30/0.1)
**P10**	P / -	*E. coli, E. tarda*	+	*E. coli* (84.30/0.0)	*E. coli* (87.30/0.2)	*E. coli* (84.50/0.1)
**P11**	P / -	*E. coli, A. baumannii, E. faecalis*	+	*P. mirabilis* (83.80/0.04)	*P. mirabilis* (86.80/0.0)	*P. mirabilis* (83.80/0.7)

^a^Multiple melting curve profiles were produced, indicating a polymicrobial infection

^**b**^HRMA, the most similar overall to reference strains

S. *aureus: Staphylococcus aureus, S. epidermidis: Staphylococcus epidermidis*, *S. haemolyticus: Staphylococcus hemolyticus, E. faecalis: Enterococcus faecalis, S. sanguinis: Streptococcus sanguinis, C. avidum: Cutibacterium avidum, F. magna*: *Finegoldia magna*, *B*. *melitensis*: *Brucella melitensis*, *E. coli*: *Escherichia coli, P. mirabilis: Proteus mirabilis, E. tarda: Edwardsiella tarda, P. aeruginosa: Pseudomonas aeruginosa, A. baumannii: Acinetobacter baumannii*

**Fig. 4:**
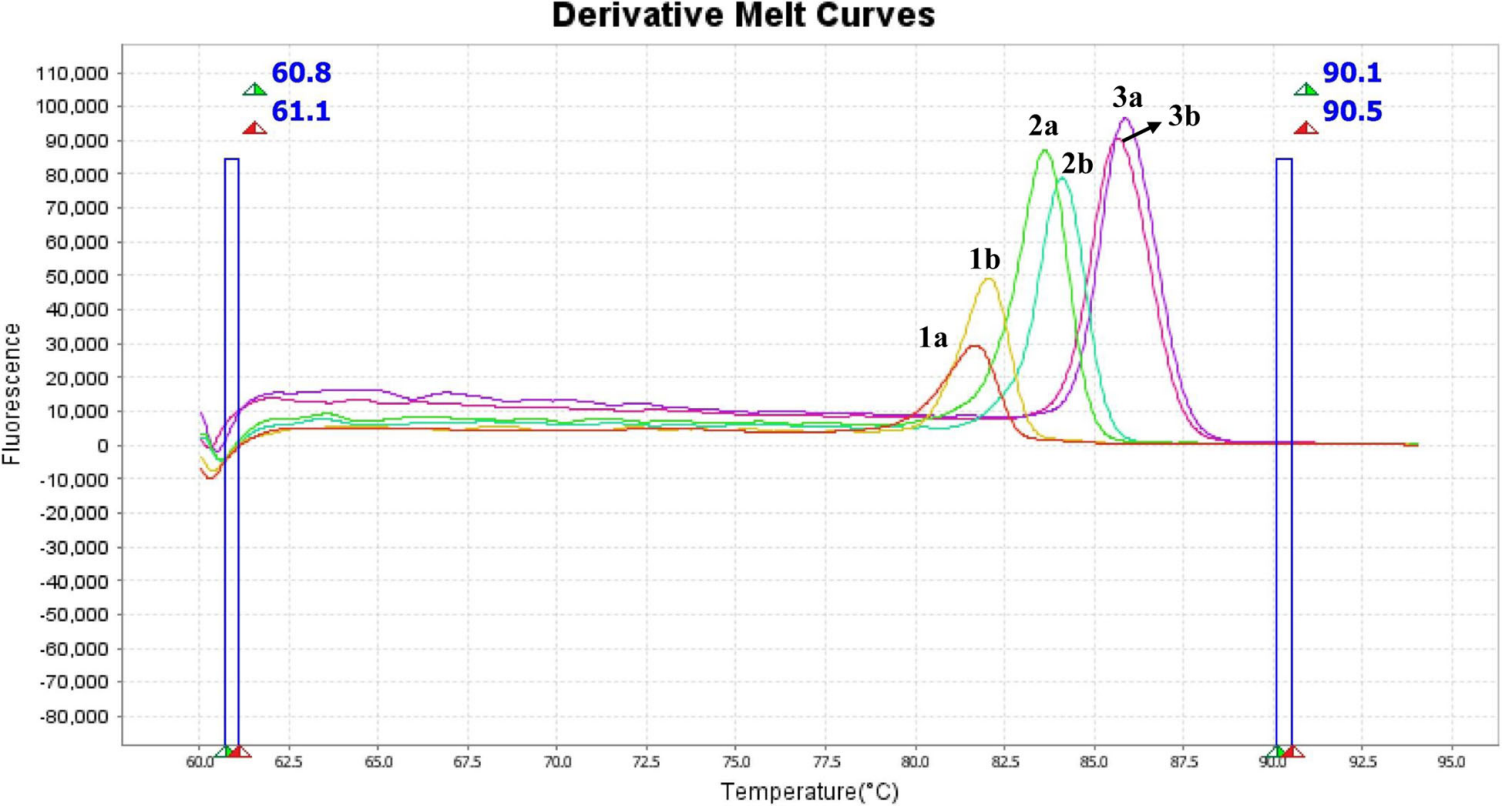
HRMA Derivative Melting Curves of Reference strains for V1, V3, V6 regions of *S. aureus* ATCC 25923 and *S. epidermidis* strain Bjg (MK 516263) **1a:** V1 region of *S. aureus*, **1b:** V1 region of *S. epidermidis*, **2a:** V6 region of *S. aureus*, **2b:** V6 region of *S. epidermidis,* **3a:** V3 region of *S. aureus*, **3b:** V3 region of *S. epidermidis*

All three gene regions of the reference strains of *S. aureus* and *S. epidermidis* outlined in Fig. 4, indicating that the two strains are distinct.

Finally, for confirmation of culture and HRMA detected strains, the sequencing method was performed.

GenBank accession numbers for the *16S rRNA* sequencing of clinical isolates determined in the present study are; *Finegoldia magna* MH201143.1, *Finegoldia magna* MK516859.1, *Staphylococcus epidermidis* MK516263.1*, Enterococcus faecalis* MK516261.1, *Corynebacterium simulans* MK516260.1, *Cutibacterium avidum* MH201146.1.

## Discussion

There are significant challenges in management of PJIs. For effective management, accurate microbiologic diagnosis of infecting organisms is important. False-negative culture and longtime for diagnosis are critical challenges for conventional methods. Molecular methods had been suggested solving these problems but their usage in routine diagnostics is still controversial.

In the present study, the prevalence of infection-causing microorganisms was determined and the role of molecular methods such as broad-range PCR and HRMA in assisting the culture method for the identification of microorganisms was investigated.

The main advantages of HRMA are the implementation of a relatively cost-effective, single-step and rapid technique for directly extracted clinical specimens and compared to other molecular methods.

The culture method was found to be able of detection of microorganisms in 74.6% (47/63) of patients as determined by the Philadelphia Consensus Criteria (PCC) and clinician’s diagnosis. This improvement can easily be explained by optimal conditions such as accurate sampling, use of various and appropriate culture media in different atmospheric conditions and prolonged incubation time. It is important to note that the culture results were positive for a significant percentage of patients who had taken antibiotics before sampling (23.4%, 11/47cases). Positive culture results may be owing to the use of optimal conditions. Of the 16 suspected culture-negative PJIs who met the consensus criteria, three cases (18.75%) receiving empirical antibiotic therapy before sampling, as expected, taking antibiotics has resulted in negative cultures.

Yoon et al. in a review study reported that the prevalence of culture-negative PJIs are up to 42% [10]. Moshirabadi et al. from Iran reported that the incidence of negative culture in PJIs are as high as 68% [20]. Kim et al. reported that the incidence of negative culture outcome was 42% in knee PJIs which was significantly higher compared to other published studies [21]. In the study of Bejon et al., frequent of culture-negative infection was 41%, and of these, 93% taking a minimum of 14 days’ antibiotic-free gap before sampling [22].

Our data show improving the culture method can be effective in increasing the positive cultures among antibiotic users, to some extent. Overall, our results revealed that the most common bacteria isolated from PJIs were Gram-positive cocci, with *S. aureus* (36.17%) and CoNS (29.78%) being the most prevalent. In most of the studies, Gram-positive cocci are involved in the majority of hip and knee PJIs. This is driven largely by infection with *S. aureus* and CoNS [23, 24]. MRS spp., especially *S. aureus* and *S. epidermidis,* will impose unnecessary antibiotic selection pressures and also, additional costs on treating patients [22]. In our evidence, the percentage of MRS isolates was 25.53% in which 10.63% *S. aureus* and 14.89% CoNS were methicillin resistance. It comprised about one-third of all cases (17/47). There is no report on the prevalence of MRS PJIs in our country and it seems that it was consistent with the prevalence of MRS in some studies [25, 26]. A study by Uckay et al. revealed 45 (75%) of the orthopedic infections caused by CoNS were MR-CoNS strains [27]. Among 47 PJIs during our study period, 11 (23.4%) cases were due to Enterococcus spp. and of these, two (4.25%) cases were related to VRE. Berbar et al. reported Enterococcus spp. accounts for only 3% of PJI [28]. In Ortega et al. study was showed that none of the Enterococcus spp. were resistant to vancomycin [29]. Our evidence revealed monomicrobial PJIs were more frequent than polymicrobial PJIs and accounted for 76.59% (36 of 47 cases) and 23.4% (11 of 47 cases, respectively. However, the prevalence of polymicrobial infection is high in our findings compared to most other studies [30, 31]. Hence, a high incidence of polymicrobial infections should be noticed in treatment of PJIs. Tan et al. reported 10.3% (108 of 1045) of the PJIs treated at their institution were polymicrobial [30]. In this study Enterococcus species, *S. aureus*, and aerobic Gram-negative bacilli, including *E. coli*, are the most frequently isolated bacteria in polymicrobial PJIs, also the frequency of other Gram- negative organisms was higher in polymicrobial PJIs than in monomicrobial PJIs. It should be mentioned that patients with a polymicrobial PJI had higher treatment failure which may be related to Gram-negative PJIs [31]. The anaerobic bacteria are identified rarely and are one of the significant causative agents of PJIs. In the present study, 6.38% (3/47) of PJIs were an anaerobic infection caused by two isolates of *F. magna* and one *C. avidum*. Khosravi et al. reported the prevalence of anaerobes in PJIs was 1.9% [32]. Soderquist et al. identified *F. magna* isolates from nine patients from 2004 to 2016 [33]. Zeller et al. revealed among 1179 PJIs treated during the study period, 15 (1%) PJIs were due to *C.avidum* which were isolated from patients with hip arthroplasty [34].

Broad-range PCR is useful for rapid reports of unknown bacterial pathogens presence in patient’s clinical specimens especially for whom are infection-suspicion and culture-negative.

The broad-range PCR specifies only bacterial DNA presence in specimens which is a disadvantage of PCR and this problem can resolve with HRMA. This study showed that broad-range PCR was able to detect bacterial DNA in 97.87% (46/47) of culture-positive samples. Also in one negative culture case, broad-range PCR was positive, therefore the results of culture and broad-range PCR were not in agreement with each other only in two cases. This finding is consistent with Rampini et al. results that showed a high concordance of 90% for broad-range PCR and culture [35]. Fifty percent concordance for cultivation and broad-range PCR was observed in Akram et al. study [3]. The results of PCR and HRMA was positive only for one negative culture case, in which *F. magna* was detected which confirmed by *16 s rRNA* gene sequencing [36].

At our experiment, *16srRNA* gene HRMA was found to be able of organism identification in 91.89% (34/37) of patients which were concordant with monomicrobial culture results (four cases were accordant at the genus level) and 8.1% (3/37) of HRMA results were discordant with culture. It is likely that these infections were polymicrobial, with only one of the pathogens was identified by culture and, the other causative agent distinguished by HRMA, due to HRMA’s inability to resolve polymicrobial infections. In confirmation of this, in 3/37 discordant samples, HRMA generated multiple melting peaks in their derivative plots. Our finding is consistent with Won et al. study that evaluated a broad-based PCR assay coupled with HRMA for bacterial identification in bacterial septic patients (52 cases). Their assay results were concordant with culture findings in 46/52 (88.5%) [37].

In our study, of 47 (mono + poly) cases, 41 (87.23%) cases were detected by HRMA. The sensitivity and specificity of total HRMA (mono + poly) vs culture were 82.92, 93.75%., respectively. Hardick et al. showed that HRMA concordance with species identification of bacterial pathogens in ascitic fluids from patients with suspected spontaneous bacterial peritonitis (mono+poly) was 70.6% and the following sensitivity and specificity for 16S PCR-HRMA compared with culture techniques were 100 and 91.5%, respectively [38].

The main reason for the decrease in the sensitivity of total HRMA in our study is the inability of HRMA method in the detection of bacteria associated with polymicrobial infections.

In the present study, multiple hypervariable regions melt curve profiles of clinical strains were compared with reference strains using the Matlab-2018b program. Nevertheless, in polymicrobial infections, HRMA generated multiple dominant peaks in the derived melting curves. The Matlab-2018b program evidently supported HRMA to correctly identified individual bacterial species from polymicrobial samples (7/11) in this study. Therefore, presence multiple melting peaks in their derivative plots can suggest the presence of multiple pathogens that aiding in antibiotic selection for suspected polymicrobial infections. Won et al. had problems with polymicrobial infections and HRMA generated multiple melting peaks in their plots accordance with our study [37]. It should be noted that the probability of sample contamination should be considered when multiple melting peaks are obtained. The appropriate programs such as the Matlab-2018b program are effective for analyzing HRMA data similar to published studies by Athamanolap et al. [39]. Finally, although the identification of bacteria by conventional methods are time-consuming, the culture method is relatively practical for the identification of bacteria and unlike most molecular methods, antibiotic susceptibility can be determined by the culture method. However, some factors such as false-negative culture, slow-growing pathogens, the formation of biofilm and antibiotic prophylaxis reduce the accuracy of the culture method in identifying bacteria. It can be concluded that molecular techniques such as broad-range PCR and HRMA are complementary to this method for organism identification. Some of the investigators used different molecular techniques such as polymerase chain reaction-restriction fragment length polymorphism (PCR-RFLP) technique and new technology of Next-Generation Sequencing (NGS) to detect PJI [12, 20]. Despite the advantages of these techniques for the identification of causative organisms in PJI, significant challenges are addressed below; the PCR-RFLP technique is a gel-based and time-consuming process and large amounts of DNA required [40]. While NGS technique is costly, it does not require isolation of a pure bacteria species [41].

According to the descriptions given about culture method and HRMA, as well as their advantages and disadvantages, culture method as a Gold standard method along with a molecular method such as HRMA, to accelerate the response in laboratory will be many helpful.

The main limitation of this study was related to PCR-HRMA which is its inability to resolve polymicrobial infections. In HRMA assay, polymicrobial PJIs can be detected with respect to multiple dominant peaks in the derivate melting curve, but the individual causative agents responsible for polymicrobial PJIs cannot be correctly diagnosed by our assay [37, 38]. Nonetheless, in the current study, three discrepant samples with monomicrobial growth by culture were observed to have multiple organisms by PCR-HRMA. These results imply either contamination or a potential benefit of PCR-HRMA than conventional cultural methods to determine the presence of polymicrobial identifications. Identifying the pathogens at an earlier time point is a significant clinical benefit and help to prompt treatment [38].

In order to resolve the problem of polymicrobial PJIs identification, the use of universal digital PCR and high-resolution melt along with developed Nanoarray is suggested [42]. In HRM assay, if the causative agents of infections are not present in reference strains library, it is not detectable by the HRMA. Consequently, we recommend a vast reference strains library. Other PCR-HRMA limitations were exchanges in DNA base pairs. Changes in Tm: approx. 0.8–1.4 °C and more were performed by exchange between DNA G: C and T: A base pairs, this range and lower than this temperature range were considered as one type of bacteria [43]. Hence, in our study, the Tm difference index of HRMA was considered 0–1.4 °C which more than this range were discrepant samples with culture findings and reference strains library. Owning to the limited range of a 96–well microtiter plate, in the Real-time PCR instruments and software, it is not able to process and comparing reference strains with clinical samples for three *16SrRNA gene* regions across all rounds, entirely. We optimized the problem with the Matlab-2018b program [39].

## Conclusion

The present evidence demonstrates HRM assay is an effective screening approach to identify the pathogens involved in PJIs especially in aiding culture method. This knowledge is revealed that the HRMA is a technique of single step, rapid and relatively cost-effective. This approach can detect DNA in different clinical specimens directly extracted from PJI samples. Data analysis using Matlab-2018b program was valuable and practical to compare between detected organisms and reference strains. Using the HRMA approach and data analysis by the Matlab-2018b, monomicrobial PJIs in agreement with culture findings gained better results than polymicrobial PJIs. Future advances in this field should focus on approaches for definitive diagnosis of the causative organisms in polymicrobial PJIs identification.

## Material and methods

### Study design and population

In this study between August 2017 and November 2019, 63 patients (42 women and 21 men) aged 41–85 years were qualified. The specimens (39 synovial fluids and 24 tissues from knee and hip joint) were taken from patients who underwent knee and hip joint surgeries in PJIs (63 cases) under observing tertiary care hospitals located in Tehran, Iran. Patients characteristics were documented according to the involved joints (hip and knee), age, gender, inflammatory markers (CRP, ESR), white blood cell, fever, antibiotic consumption (in ≤ 2 to 4 weeks preceding surgery) [21].

### Case definition

The cases included in this study belonged to the PJIs (63 cases). The patients who had one or two major criteria for PJI based on PCC, the first, two positive cultures of the same pathogens or the second, a sinus tract in association with the joint - and/or had two or three of the following minor criteria - raised up serum CRP and ESR (with abnormal values of **>** 10 mg/ml and **>** 30 mm/h, respectively), raised up synovial leukocyte count or raised up synovial neutrophil percentage (with abnormal values and percentage of **>** 1700 cell/μl and **>** 65% respectively, for knees and **>** 3000 cells/μl and > 80%, respectively, for hips were considered abnormal) were considered as cases of PJI [20, 21, 44–46].

### Microbiological culture and biochemical testing approaches

The synovial fluid and tissue specimens were taken during aspiration and knee and hip joint surgery. Specimens were cultured on both of the broth (such as thioglycolic acid, brain heart infusion broth, and resin-containing BACTEC Blood Culture Media) and agar medium (such as brain heart infusion, blood, chocolate, MacConkey, and schaedler agar supplemented with Vitamin K1) in 37 °C for at least 48 h under aerobic, microaerophilic (with 5–7% CO2) and anaerobic conditions. The incubation period was extended to 14–21 days for slow-growing and fastidious microorganisms. The identification of isolated organisms was done by biochemical tests [47] and were confirmed with VITEK 2 Compact system (BioMerieux, USA). Vancomycin minimum inhibitory concentrations were determined by the E-test method (MIC test strip, liofilchem, Italy) for MRS ssp. Additionally, VRE was determined using the disk diffusion test.

### Extraction of nucleic acids

DNA extraction of 63 specimens (For each patient, one synovial fluid specimen was included and tissue specimen was included for patients who had no synovial fluid) was done by Qiagen kit (QIAamp DNA Mini Kit, Germany). The quality and quantity of the extracted DNA were assayed by spectrophotometry at 260 and 280 nm UV-vis light. Extracted DNA was stored at − 20 °C for the PCR and HRMA [20].

### Broad-range PCR

The *16SrRNA* PCR of 63 specimens was achieved by universal primers (27F: AGAGTTTGATCCTGGCTCAG, 1429R: GGTTACCTTGTTACGACTT) [35]. PCR reactions were performed in 25 μl final concentration: 10 μl of the master mix, 0.5 μl of 25 pmol F primer, 0.5 μl of 25 pmol R primer, 12 μl sterile distilled water and 2 μl genomic DNA. PCR conditions were 95 °C for 300 s, followed by 37 cycles of 95 °C for 45 s, 58 °C for 30s (annealing temperature), 72 °C for 70s and final extension 72 °C for 300 s [14].

### The HRMA of *16S rRNA* gene hypervariable regions

The *16S rRNA* HRMA (Applied Biosystems Step OnePlus Systems, USA) was performed for samples with positive broad-range PCR (48 synovial fluid and tissue samples) and of these, one sample was with a positive culture and negative broad-range PCR.

At first, HRMA was done with extracted DNA of 19 species of bacteria (reference strains) which are associated with PJIs. By targeting V1, V3, and V6 hypervariable regions, each of species had a specific melting curve group consist of three melting curves. Finally, a melting curve library was prepared to depend on reference strains. In the following, HRMA was done with extracted DNA of synovial fluid and tissue samples and then melting curve data generated from each of the samples were compared to the melting curve library of reference strains [14, 21, 48]. Reference strains used in this study were *S. aureus* ATCC 29213, *S. aureus* ATCC 25923*, S. epidermidis* strain Bjg (MK 516263), *S. hemolyticus* ATCC 29970, *S. saprophyticus* ATCC 15305, *Micrococcus luteus* ATCC 15307, *Enterococcus faecalis* ATCC 29212, *Streptococcus sanguinis* ATCC 10566, *Finegoldia magna* ATCC 1766, *Cutibacterium* ATCC 25573*, Haemophilus influenza* ATCC 49766, *Pseudomonas aeruginosa* 27,853, *Escherichia coli* ATCC 25922, *Klebsiella pneumonia* ATCC 700603, *Proteus mirabilis* ATC 43071, *Acinetobacter baumannii* ATCC 19606, *Brucella melitensis* 16 M ATCC 23456, *Mycobacterium tuberculosis* H37Rv, *Corynebacterium simulans* strain BJd (MK 516260).

HRMA reactions were performed in a 20 μl final volume and contained the following final concentrations: 4 μl of 5x EvaGreen master mix (5x HOT FIREPol EvaGreen HRM Mix by Solis BioDyne), 0.6 μl of 10 pmol F primer, 0.6 μl of 10 pmol R primer (Table 2), 13.8 μl sterile deionized water and 1 μl genomic DNA (each of three primer pairs were in three reactions, individually). PCR and HRMA conditions were 95 °C for 300 s, followed by 40 cycles of 95 °C for 10s, 58 °C for 30s (annealing temperature), and 72 °C for 10s and melt curve stage with 95 °C for 15 s, 60 °C for 60s, 95 °C for 15 s. Some of the HRMA-positive samples were confirmed by DNA sequencing and recorded in the nucleotide database in NCBI.

**Table 2:** Primers targeting the bacterial *16S rRNA* gene for HRMA

Region	Primer	Sequence (5′-3′)	Product size (bp)	Ref
**V1**	**V1-F** **V1-R**	GYGGCGNACGGGTGAGTAA TTACCCCACCAACTAGC	170–171	[49]
**V3**	**V3-F** **V3-R**	CCAGACTCCTACGGGAGGCAG CGTATTACCGCGGCTGCTG	204–205	
**V6**	**V6-F** **V6-R**	TGGAGCATGTGGTTTAATTCGA AGCTGACGACANCCATGCA	128–131	

### Processing for HRMA results by Matlab-2018b program

To assay HRM, experimentally produced reference strains and clinical isolates melting curves (raw melting data files) were exported from Step One software v2.3 and imported into Matlab-2018b program, where the melting curves were first translated with a translation function and afterward compared with the melting curve database produced in reference strains in each hypervariable region of *16srRNA* gene (V1, V3, V6), individually. A reference library based on Tm and melting curves of 19 reference strains was established for the identification of pathogens. The Matlab program consists of three main parts, reading data from exported raw data files and preprocessing, analyzing data and assigning most similar reference strains to each clinical isolate, and writing the answers to the file and visualizing.

In the beginning, the program was written with the relevant codes and was executed by Matlab-2018b then was classified as different clinical isolates and reference strains. In the following, their melting curves and Tm were compared to each other for organism’s identification [39]. Herein, programming, processing, and analyzing were performed by the designed program based on hypervariable regions of the *16srRNA* gene with temperature range between 80.90 °C-89.90 °C (based on obtained reference strains Tm). Detection criteria in Matlab-2018b analysis are based on a comparison of reference strains to correspond Tm with the closest clinical isolate Tm (for every three regions of the gene with different primers, individually). Indeed, Tm differentiation distances between the reference strains and clinical isolates were indicated with an aggregated difference index (0 °C-1.4 °C) [43]. Tm peaks belong to *16srRNA* V1, V3 and V6 regions in reference strains and clinical strains were compared with each other in this program, and the final results were taken according to the closet similarity between these two groups.

In our study, sensitivity, specificity, PPV, NPV, and accuracy were calculated for HRMA vs culture based on the PCC definition of PJIs.

## Data Availability

All documents and additional data are available from the corresponding author upon reasonable request.

## Abbreviations

PJI: Periprosthetic joint infection
PCC: Philadelphia Consensus Criteria
HRMA: High-Resolution Melt Analysis
Tm: Melting temperature
PPV: Positive predictive values
NPV: Negative predictive values
*S. aureus*: *Staphylococcus aureus*
CoNS: Coagulase-negative staphylococci
*F.magna*: *Finegoldia magna*
*C. avidum*: *Cutibacterium avidum*
MRS spp.: Methicillin-resistant *Staphylococcus* spp.
MRSA: Methicillin-resistant *Staphylococcus aureus*
MRSE: Methicillin-resistant *Staphylococcus epidermidis*
VRE: Vancomycin-resistant enterococci
